# Study on the Heat Source Insulation of a Thermal Bubble-Driven Micropump with Induction Heating

**DOI:** 10.3390/mi12091040

**Published:** 2021-08-29

**Authors:** Bendong Liu, Chenxu Ma, Jiahui Yang, Desheng Li, Haibin Liu

**Affiliations:** 1 Faculty of Materials and Manufacturing, Beijing University of Technology, Beijing 100124, China; machenxu.bjut.edu.cn@emails.bjut.edu.cn (C.M.); klrs80@sohu.com (J.Y.); dsli@bjut.edu.cn (D.L.); liuhb@bjut.edu.cn (H.L.); 2 Electrical and Mechanical College, Beijing Vocational College of Agriculture, Beijing 102208, China

**Keywords:** heat source insulation, thermal bubble driven, micropump, microfluidics, induction heating

## Abstract

Thermal bubble-driven micropumps have the advantages of high reliability, simple structure and simple fabrication process. However, the high temperature of the thermal bubble may damage some biological or chemical properties of the solution. In order to reduce the influence of the high temperature of the thermal bubbles on the pumped liquid, this paper proposes a kind of heat insulation micropump driven by thermal bubbles with induction heating. The thermal bubble and its chamber are designed on one side of the main pumping channel. The high temperature of the thermal bubble is insulated by the liquid in the heat insulation channel, which reduces the influence of the high temperature of the thermal bubble on the pumped liquid. Protypes of the new micropump with heat source insulation were fabricated and experiments were performed on them. The experiments showed that the temperature of the pumped liquid was less than 35 °C in the main pumping channel.

## 1. Introduction

Micropumps are the driving source and important components of Microfluidic systems [1]. Depending on the actuation mode, micropumps can be classified into piezoelectric [2], electrostatic [3,4], electromagnetic [5], electroosmotic [6,7], electrochemical [8] and thermal bubble-driven micropumps [9,10,11]. Due to having no mechanical moving parts, no friction and rigid impact, thermal bubble-driven micropumps have advantages including high reliability, simple structure and simple fabrication process.

Generally, thermal bubble-driven actuators use an electric resistive heater to generate heat and thermal bubbles [12,13]. This driving method has been widely used in ink-jet printing [14,15]. Recently, many studies of thermal bubble-driven micropumps have been conducted. Tsai and Lin [16] designed a thermal bubble-driven micropump with an aluminum resistive heater. Jung and Kwak [17] developed a thermal bubble-driven micropump using an embedded polysilicon microheater.

In the early stage, our group studied a high flow rate thermal bubble-driven micropump with induction heating [18] The micropump is mainly composed of a glass substrate, an excitation coil, a metal heating plate and a PDMS (polydimethylsiloxane) chip. The micro metal heating plate is located in the pump chamber. The external excitation coil is outside of the pump chamber, which provides energy to the micro heating plate via the electromagnetic field. When an eddy current is generated in the heating plate, the temperature of the heating plate rises rapidly. A small amount of liquid in contact with the micro heating plate is vaporized to produce several large-volume thermal bubbles. Pumping is realized by the periodic expansion and contraction of the thermal bubbles. The pumping flow rate can reach about 102 μL/min. All thermal bubble-driven micropumps of the type mentioned above function by the thermal bubble directly driving the pumped liquid. The thermal bubble will damage some biological or chemical properties of the solution due to the high temperature of the thermal bubble and the direct contact with liquid. For example, half of the enzyme activity of subtilis is lost at a temperature of 52.8 °C. Similarly, half of the enzyme activity of glutinis wheat germ is lost at a temperature of 56.5 °C. The safe temperature is about 40 °C for most of enzymes according to the research of Daniel [19].

In order to reduce the influence of the high temperature of the thermal bubbles on the pumped liquid in the thermal bubble-driven micropump, this paper proposes a kind of heat insulation micropump driven by thermal bubbles with induction heating. Induction heating with high frequency alternating current (AC) was adopted and the directional driving of the micro fluid was realized with the periodic expansion and contraction of thermal bubbles in the design. In addition, the heat source of the thermal bubble was insulated from the pumped liquid to avoid the high temperature damage to biological or chemical substances in the solution. Therefore, the new micropump studied in this paper has the advantages of small heat influence of thermal bubble on the pumped liquid, heat source insulation, simple structure and easy integration.

## 2. Design and Working Principle

### 2.1. Design

**Figure 1:**
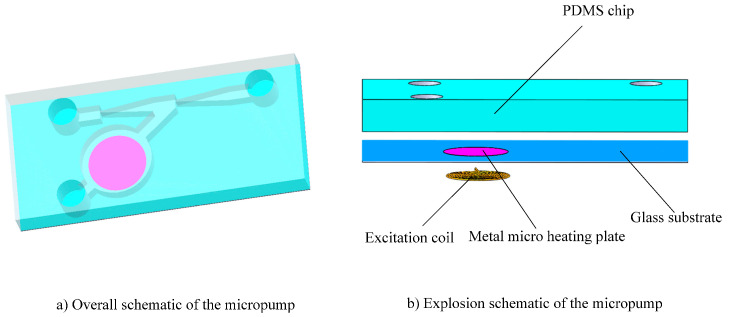
Schematic of the heat source insulation micropump. (**a**) Overall schematic of the micropump; (**b**) Explosion schematic of the micropump.

The structure diagram of the heat source insulation micropump is shown in Figure 1. The pump is mainly composed of a PDMS chip, a metal micro heating plate, an excitation coil and a glass substrate.

**Figure 2:**
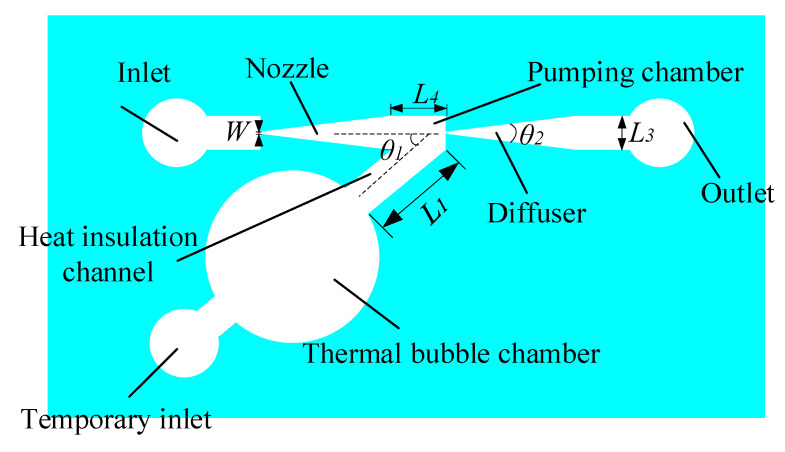
Schematic drawing of the PDMS chip of the heat source insulation micropump.

The micro heating plate was designed to sit on the top surface of the glass substrate and the excitation coil was designed to adhere on the bottom surface of the glass substrate The other structures of micropump were designed to be made in a PDMS chip. The schematic drawing of the PDMS chip of the heat source insulation micropump is shown in Figure 2. The structure includes an inlet, a temporary liquid inlet, an outlet, a thermal bubble chamber, a heat insulation channel, a pumping chamber and a diffuser/nozzle. The power of the thermal driven micropump is provided by the expansion and shrinkage of the thermal bubbles. Thermal bubbles will be generated when the temperature of the micro heating plate reaches the nucleation temperature. However, the high temperature of the thermal bubbles can damage something in the liquid if the liquid is too close to the thermal bubbles.

In this paper, the thermal bubble and its chamber was designed on one side of the main pumping channel. The main pumping channel is composed of the nozzle, the diffuser and the pumping chamber. The pumped liquid flows from the inlet to the outlet through the main pumping channel. The thermal bubble chamber connected with the main pumping channel of the micropump through a side heat insulation channel. The high temperature of the thermal bubble is insulated from the pumped liquid by the liquid in the heat insulation channel, which can reduce the influence of the high temperature of the thermal bubble on the pumped liquid.

The diameter of the thermal bubble chamber of the heat source insulation micropump is 5 mm. The angle between the heat insulation channel and the main pumping channel, *θ*_1_, is 40°. A pair of nozzle and diffuser flow controllers was designed with an 80 μm width at the narrow neck *W*, 1 mm at the open mouth and the diverging angle, *θ*_2_, is 14° [16]. The rectangle pumping chamber is 1.5 mm in length (*L*_4_) and 1 mm in width (*L*_3_). The length of the heat insulation channel (*L*_1_) is 2.5 mm and the depth of all micro channels is 150 μm. The diameters of the inlet, outlet and temporary inlet are 2 mm. The temporary liquid inlet is used to inject liquid into the bubble chamber before pumping, after which it is plugged with a cylindrical PDMS plug.

We simulated the liquid velocity with finite element Multiphysics software (COMSOL Inc., Palo Alto, CA, USA). In our simulation, *θ*_1_ was gradually increased from 30° to 75°. The results show that the flow velocity in the main pumping channel was fastest when *θ*_1_ was about 40°. As a result of that, *θ*_1_ is selected as 40°.

### 2.2. Working Principle

**Figure 3:**
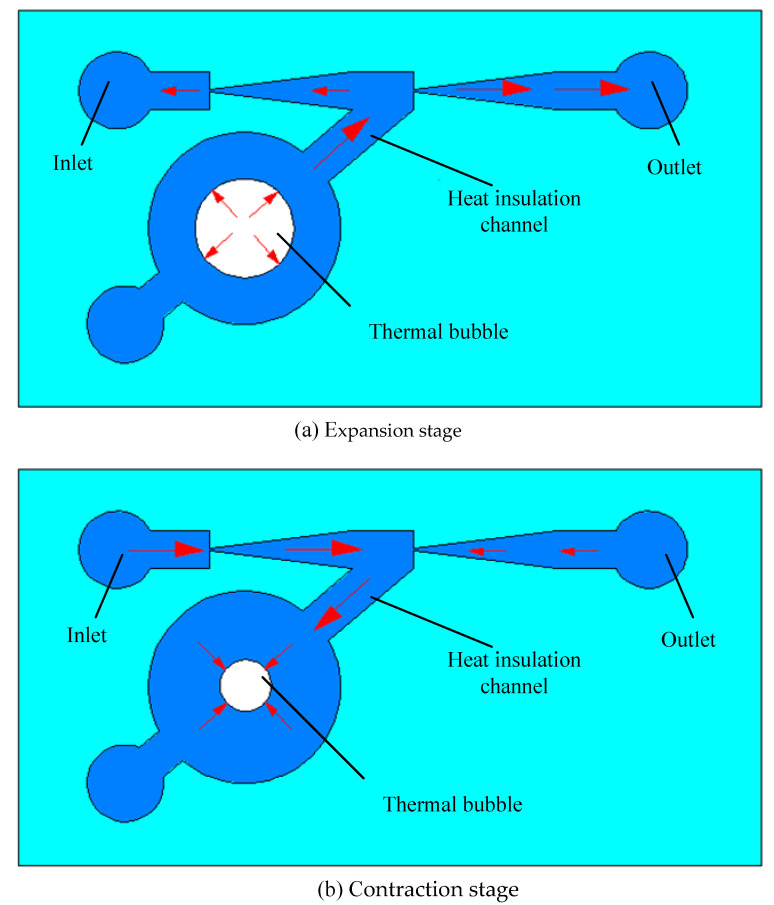
Working principle of the heat source insulation micropump. (**a**) Expansion stage; (**b**) Contraction stage.

The working principle of the heat source insulation micropump with induction heating is shown in Figure 3. When high frequency alternating current is applied to the excitation coil, an alternating magnetic field will be generated around the coil. Under the action of the alternating magnetic field, an eddy current will be generated in the metal micro heating plate, and consequently, heat will be generated in the micro heating plate. Then, the liquid on the surface of the micro heating plate is heated through heat conduction. When the liquid reaches the nucleation temperature, thermal bubbles will appear on the surface of the micro heating plate. As shown in Figure 3a, with the rapid growth of the thermal bubble, the pressure in the thermal bubble chamber increases quickly. Under the effect of the bubble pressure, the liquid flows rapidly to the heat insulation channel. After that the liquid flows into the pumping chamber through the heat insulation channel. Then the liquid in the pumping chamber will flow into the nozzle and the diffuser, simultaneously. Due to the different flow resistance in two directions, the volume of liquid flowing into the direction of the diffuser and the liquid outlet is greater than that flowing in the nozzle direction.

If the high frequency alternating current applied to the excitation coil is powered off, the micro heating plate stops heating and the thermal bubbles will shrink, cooling with the surrounding environment, and the pressure in the thermal bubble chamber decreases. Then the liquid flows back from the main pumping channel to the thermal bubble chamber through the heat insulation channel. At the same time, both the liquid of inlet and the liquid of outlet flow into the heat insulation channel through the nozzle and the diffuser, respectively. Due to the different flow resistance in the two directions, more liquid flows into the heat insulation channel from the inlet through the nozzle than that from the outlet through the diffuser, as shown in Figure 3b.

Therefore, in a pumping cycle, there will be a certain net flow from the inlet to the outlet. The periodic expansion and contraction of thermal bubbles can realize the pumping function of the heat source insulation micropump driven by thermal bubbles.

The temperature of the liquid close to the thermal bubbles decreases gradually. The liquid with high temperature moves back and forth in the heat insulation channel with the periodic expansion and contraction of the thermal bubbles. As long as the high temperature liquid cannot enter the main pumping channel in the expansion stage of the thermal bubbles, the liquid pumped in the main channel will not be affected by the high temperature of the thermal bubbles.

When the thermal bubble expands periodically, its volume increases from the minimum to the maximum, and the volume of liquid driven by it is equal to its volume variability. The volume variability of thermal bubbles can be calculated with Equation (1).

(1) $$V_{1}=\frac{4}{3}\pi (R_{2}{}^{3}-R_{1}{}^{3})$$

Here, $R_{2}$ is the equivalent diameter of the maximum thermal bubble, $R_{1}$ is the equivalent diameter of the thermal bubble after shrinkage.

Therefore, as long as the volume of the heat insulation channel is greater than the variability of the bubble volume, the high temperature liquid will not enter the main channel. The equivalent volume of the insulation channel can be calculated with Equation (2)

(2) $$V_{2}=L_{3}bc$$

where $L_{3}$ is the width of the heat insulation channel, b is the equivalent length of the heat insulation channel in the micropump and c is the depth of the heat insulation channel.

Assuming that the equivalent thermal bubble increases from 100 µm to 500 µm in diameter and the thermal bubble grows in the center of the thermal bubble chamber, the length of the thermal insulation channel is 2.5 mm, the radius of the thermal bubble chamber is 2.5 mm and the equivalent length of the heat insulation channel is about 4.5 mm. According to Equation (1), the volume variability of the thermal bubbles is about 0.52 mm^3^. According to Equation (2), the total volume of the equivalent insulation channel is about 0.68 mm^3^. When the diameter of the thermal bubbles increases from 100 µm to 500 µm, the high temperature liquid will not flow into the main channel.

The mean output flow rate per second can be calculated with Equation (3) [17,20].

(3) $$Q=\frac{2\Delta V_{m}}{T}\frac{\sqrt{\frac{19}{20}(\frac{L_{3}}{W}}{)}^{0.34}-1}{\sqrt{\frac{19}{20}(\frac{L_{3}}{W}}{)}^{0.34}+1}$$

Here, $\Delta V_{m}\;$is the maximum volume variability induced by the thermal bubble, W is the neck of the diffuser/nozzle structure, $L_{3\;}$is the mouth of the diffuser/nozzle structure. T is one period of the micropump.

**Figure 4:**
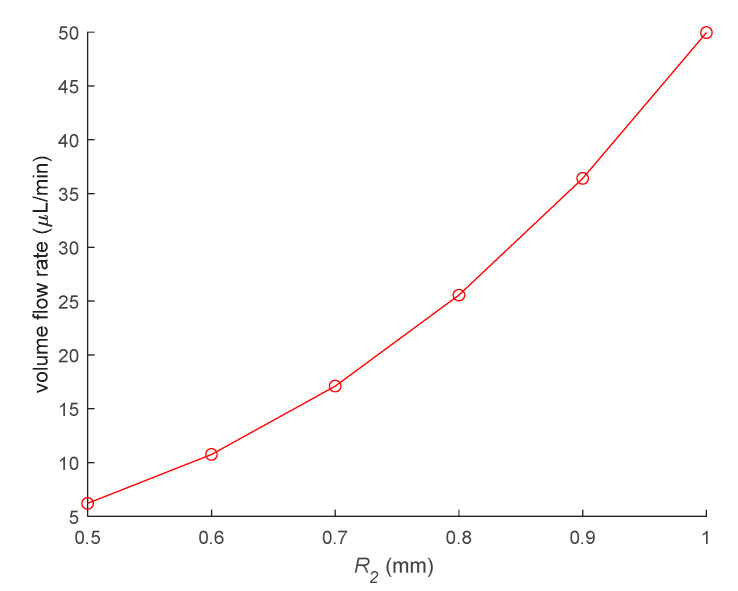
Theoretical volume flow rate of the micropump.

Assuming that the diameter of the thermal bubble after shrinkage is 100 µm, when the maximum diameter of the thermal bubble increases from 500 µm to 1000 µm, the theoretical volume flow rate is shown in the Figure 4.

## 3. Fabrication

**Figure 5:**
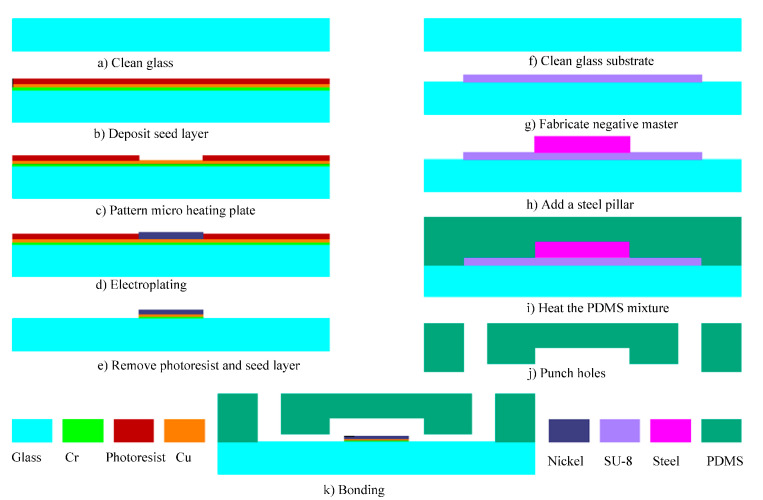
Fabrication process of the thermal bubble-driven micropump. (**a**) Clean Class; (**b**) Deposit seed layer; (**c**) Pattern micro heating plate; (**d**) Electroplating; (**e**) Remove photoresist and seed layer; (**f**) Clean glass substrate; (**g**) Fabrication negative master; (**h**) Add a steel pillar; (**i**) Heat the PDMS mixture; (**j**) Punch holes; (**k**) Bonding.

The schematic of the fabrication process involved in the realization of the thermal bubble-driven micropump is shown in Figure 5 using traditional lithography and electroplating. Firstly, a glass slide of 200 µm in thickness was cleaned as shown in Figure 5a. Cr and Cu layers were deposited as the seed layer on the glass substrate, then positive photoresist (BP212) was spun on the top of Cr/Cu layers Figure 5b and the Cr/Cu were patterned to define the micro heating plate Figure 5c. The micro heating plate was electroplated at 50 °C in a low stress nickel sulfamate bath [21] at a current density of 2 A/dm^2^, resulting in the Ni micro heating plate with a thickness of 20 µm (Figure 5d). After that, the photoresist was removed with acetone, the Cr/Cu seed layers were removed with a solution of hydrochloric acid and glycerol and a solution of ferric trichloride and water (weight ratio of 1:20), respectively, as shown in Figure 5e.

The PDMS chips were fabricated using conventional soft-lithography techniques. Briefly, a glass substrate was cleaned (Figure 5f) and a negative master was fabricated on the glass substrate with SU-8 photoresist (SU-8 2050, Micro-Chem Corp, Newton, MA, USA) using a transparency mask and a mask aligner (BGT-3B, Beijing Chuangweina Technology Co. Ltd., Beijing, China) Figure 5g. A steel pillar of 5 mm in diameter and 2 mm in height was added, aligned with the thermal bubble chamber as shown in Figure 5h.

**Figure 6:**
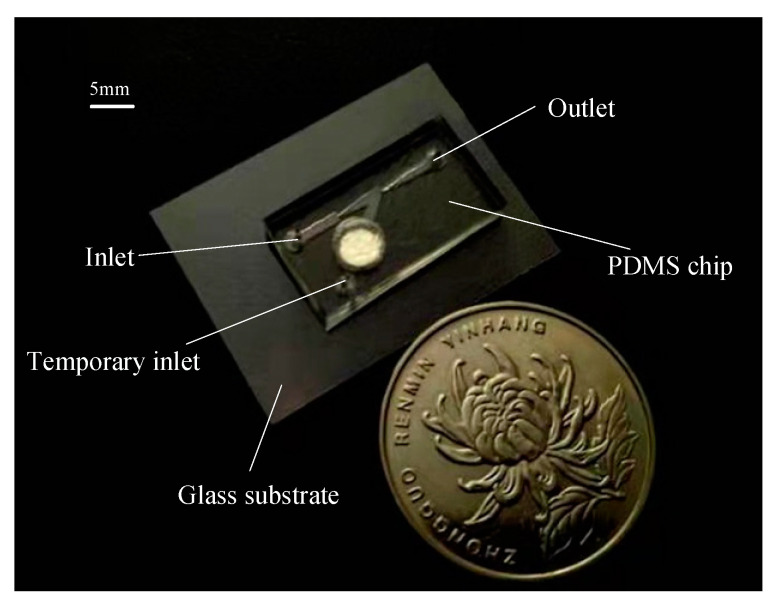
Photograph of one thermal bubble-driven micropump.

A prepolymer of PDMS (Sylgard 184, Dow Corning, Midland, MI, USA) and curing agent was thoroughly mixed at a ratio of 10:1 (*wt*/*wt*) and the PDMS mixture was degassed in a vacuum chamber for 30 min. The PDMS mixture was carefully poured into the SU-8 master mold and then cured at 60 °C on a heating plate for 2 h as shown in Figure 5i. Then the PDMS chip, with a thickness of 3 mm, was peeled off from the mold and three holes with a radius of 1 mm were punched in the PDMS replicas to allow for the connection of tubes used as the inlet and the outlet as shown in Figure 5j. The fabrication was followed by oxygen plasma treatment for irreversible bonding between the PDMS chip and the glass substrate with the micro heating plate as shown in Figure 5k. A photograph of one protype of the thermal bubble-driven micropump with heat source isolation is shown in Figure 6. Before experiment, a 16-turn planar spiral coil was fabricated from copper enameled wires with a diameter of 80 μm and was glued to a PCB board under the micro heating plate.

## 4. Measurement of the Flow Rate and the Back Pressure

The control system of the thermal bubble-driven micropump with heat insulation is the same as in our previous study [22]. High frequency alternating current was supplied with a high frequency pulse generator (SP1631A, Nanjing Nanjingshengpu Technology Co. Ltd., Nanjing, China). The current was applied to the excitation coil of the micropump through a relay (HSIN DA, 943-1C-5DS, Taiwan Xinda Precision Co., Ltd. Changzhou, China). The on/off sequence of the relay was controlled by a programmable controller (MITSUBISHI, MELSEC FX2N-48MT, Mitsubishi Electric Corporation, Tokyo, Japan).

**Figure 7:**
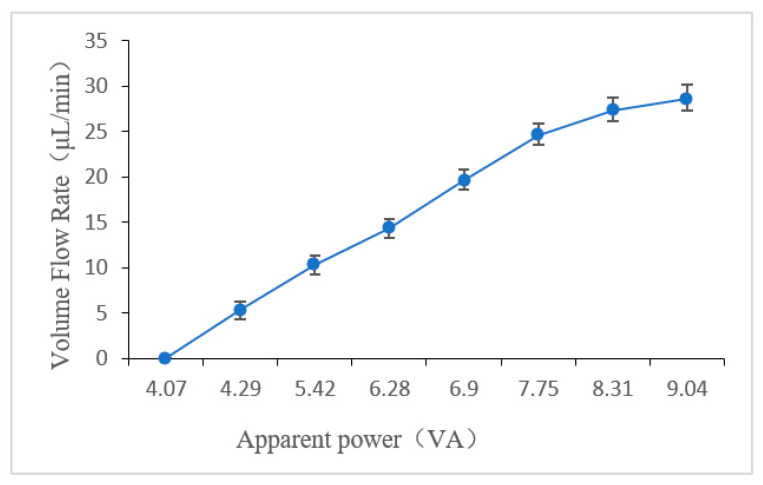
Pumping flow rate VS apparent power.

In our experiments, the frequency of the alternating current was 80 kHz, both the heating period and the condensation period were 1 s, the applied apparent power was increased from 0 VA to 10.1 VA. The volume flow rates are the means of five measurements at each condition. Figure 7 shows volume flow rate versus the apparent power of the thermal bubble-driven micropump with heat source insulation. When the apparent power is greater than 4.07 VA, the pumping flow rate of the heat insulation micropump increased gradually with the increase in the power, and the maximum flow rate of the micropump was about 30 μL/min. When the apparent power was greater than 10.01 VA, the heat generated by the micro heating plate was too much, and the heat cannot be transferred out of the thermal bubble chamber during the condensation period, which will lead to the insufficient contraction of the thermal bubbles. Therefore, the volume change of thermal bubble is too small, and the pumping flow rate will be significantly reduced. When the applied apparent power was 9.04 VA, the maximum back pressure of the micropump was about 118 Pa.

Table 1 lists the main performance and related parameters of the thermal bubble-driven micropump described here and some others from the literature. It can be seen that the micropump with induction heating has a larger pumping flow rate. The back pressure of the micropump with induction heating is lower compared to the micropump with resistance heating due to lower working frequency and larger dimensions of diffuser/nozzle structure. It is noted that the pumping flow rate and the back pressure of the heat insulation thermal bubble-driven micropump are lower than the thermally driven micropumps with induction heating but without heat insulation. The power that is used for heating is about half of the apparent power. Due to the added channel, the flow resistance is increased accordingly and the speed of the liquid that passes through the diffuser/nozzle structure is deceased. Accordingly, the function of the check valve of the diffuser/nozzle structure is decreased, and the pumping flow rate and the back pressure of the heat insulation thermal bubble-driven micropump also decrease.

## 5. Fluorescence Temperature Measurement

The temperature measurement using fluorescence intensity changes is a non-contact temperature measurement technology [23,24] that has the advantages of low requirements for measuring instruments, fast response, high resolution, high sensitivity and large temperature measurement range [25,26]. Therefore, the effect of the heat insulation of the micropump was measured with using fluorescence intensity.

### 5.1. Experimental Setup of Fluorescence Temperature Measurement

**Figure 8:**
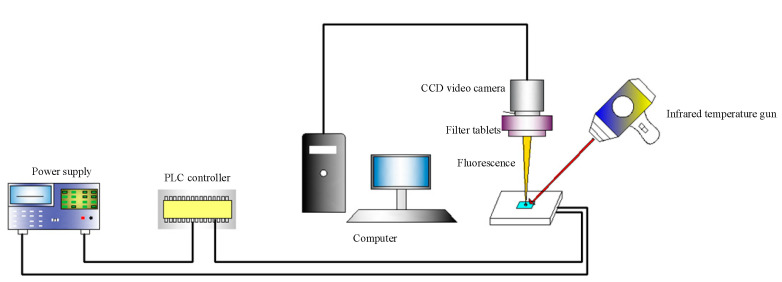
Schematic of fluorescence temperature measurement.

An experimental setup of fluorescence temperature measurement with microscope was built as shown in Figure 8. The fluorescence excitation of a fluorescence microscope (DSY5000X, Chongqing Aopu Photoelectric Technology Co., Ltd, Chongqing, China) was selected as the light source, and Rhodamine B was selected as the fluorescent dye. Rhodamine B was dissolved in the deionized water to form a Rhodamine solution with a concentration of 0.02 mol/L. Rhodamine B solution was irradiated with the fluorescence microscope to inspire fluorescence. The temperature of the solution was measured in real time with an infrared thermometer (TM910, Taikeman Technology Co. Ltd., Shenzhen, China), and the fluorescence image of the solution in the microchannel at this temperature was saved to the computer. In order to reduce the experimental error, four fluorescence images of each measurement at the same temperature were saved. Then the fluorescence images were grayed according to the fluorescence intensity, corresponding to the temperature.

The Rhodamine B solution at 21 °C was selected as the standard low temperature and the solution at 90 °C was selected as the standard high temperature. Then, we normalized the fluorescence intensity corresponding to its temperature according to Equation (4)

(4) $$I_{n}=\frac{I-I_{\mathrm{high}}}{I_{\mathrm{low}}-I_{\mathrm{high}}}$$

were $I_{n}\;$is the normalized fluorescence intensity, I is the fluorescence intensity extracted from the grayed fluorescence image, $I_{\mathrm{high}}\;$is the fluorescence intensity of the standard high temperature (90 °C) and $I_{\mathrm{low}}\;$is the fluorescence intensity of the standard low temperature (21 °C).

**Figure 9:**
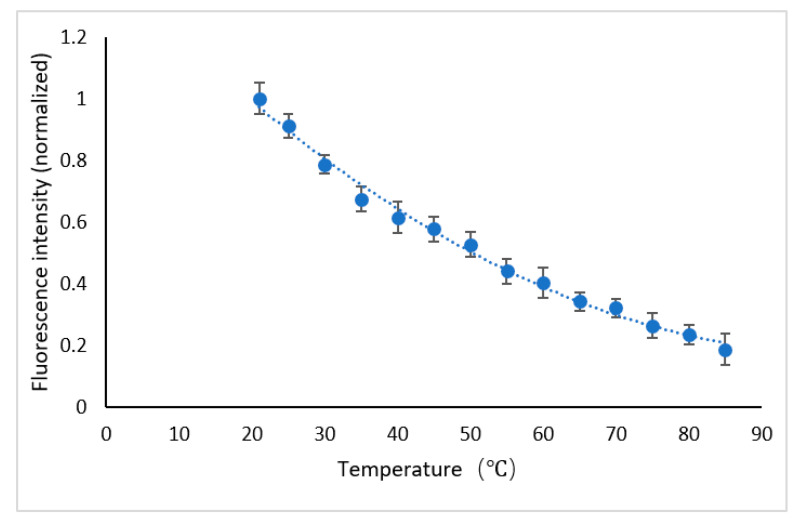
Relationship between fluorescence intensity and temperature.

The curve fitting between temperature and normalized fluorescence intensity was carried out with the binomial fitting method. The fitting formula is shown as Equation (5)

(5) $$y=0.001x^{2}-0.0247x+1.4374$$

where x is temperature and y is the fluorescence intensity after normalization. Figure 9 shows the relationship between the fluorescence intensity and the temperature after normalization. It can be seen that the fluorescence intensity decreases with the increase in temperature.

### 5.2. Measurement of Heat Insulation

In order to test the heat insulation effect of the thermal bubble-driven micropump, the temperature of the solution in the main pumping channel and in the heat insulation channel of the micropump were measured.

**Figure 10:**
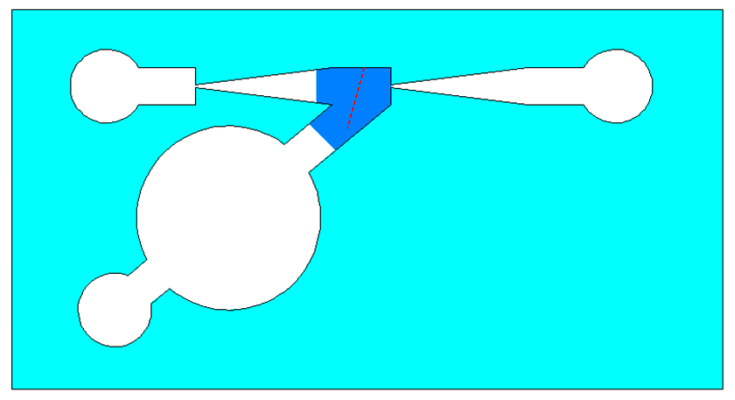
Schematic of temperature measurement area.

**Figure 11:**
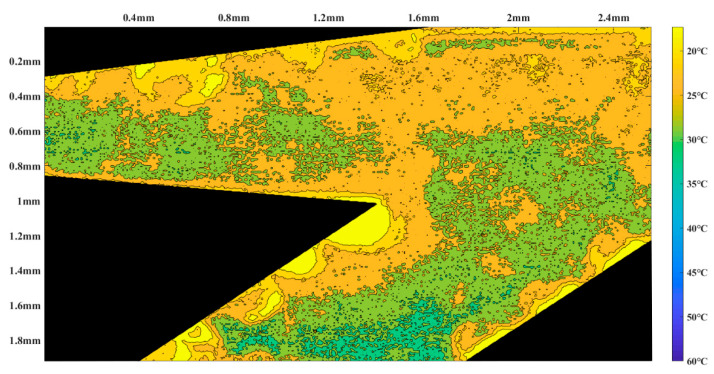
Temperature nephogram in the thermal bubble expansion stage.

**Figure 12:**
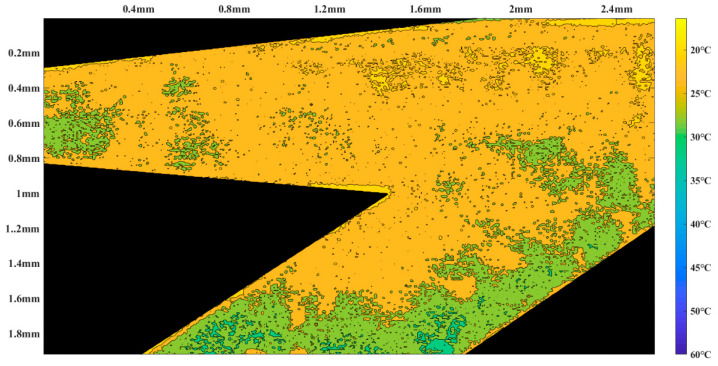
Temperature nephogram in the thermal bubble contraction stage.

Firstly, Rhodamine B solution with a concentration of 0.02 mol/L was introduced into the micropump from the temporary liquid inlet to keep rhodamine B solution filling the thermal bubble chamber and the heat insulation channel. Then, the temporary inlet was blocked, and the Rhodamine B solution with the same concentration was injected into the main pumping channel of the micropump from the inlet. In order to keep zero back pressure in the pumping process, the horizontal tubes that connected with the inlet and the outlet were kept at the same height. Alternating current with frequency of 80 kHz was applied to the excitation coil, both the induction heating time and the interruption time were 1 s. The temperature measurement area was some part of the micropump, which is shown as the blue area in Figure 10. According to the fitting relationship between fluorescence intensity and temperature, the temperature distribution of the region is obtained when the fluorescence intensity of the solution is measured in the micropump. Then, the region was drawn as a temperature nephogram according to the fluorescence intensity. Figure 11 and Figure 12 are partial temperature nephograms of the heat insulation channel and main pumping channel in the thermal bubble expansion stage and in the thermal bubble contraction stage, respectively, with an applied apparent power of 6.28 VA. From the temperature distribution nephogram, it can be seen that the liquid temperature in the heat insulation channel was higher than that in the main pumping channel. The temperature of the solution in the main pumping channel was lower than 35 °C even in the expansion stage at apparent power of 6.28 VA. It is clear that the temperature of the thermal bubble is higher than 100 °C and the solution near to the thermal bubble also has a high temperature. The temperature in the main pumping channel is safe for most chemical and biological solutions with the new thermal bubble-driven micropump.

**Figure 13:**
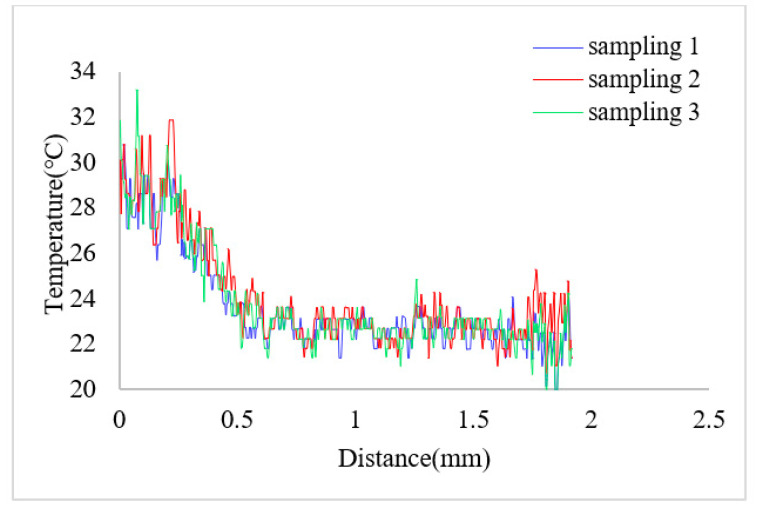
Temperature variation along the mark line (in Figure 10) when the applied apparent power is 4.29 VA.

**Figure 14:**
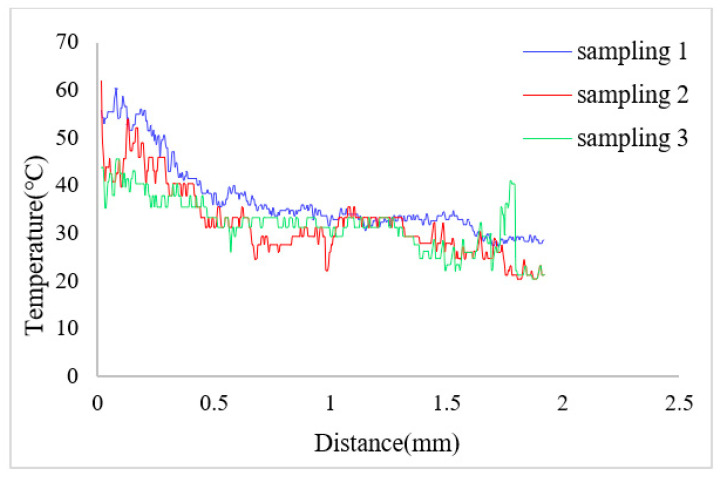
Temperature variation along the mark line (in Figure 10) when the applied apparent power is 6.28 VA.

The change of the solution temperature from the bottom to the upper along the marked line was drawn after measuring the solution temperature in the heat insulation channel and the main pumping channel. The position of the marked line is shown as the red dotted line in Figure 10. Figure 13 shows the temperature curve along the marked line at the thermal bubble expansion stage when the apparent power was 4.29 VA; the temperature difference between the heat insulation channel and the main pumping channel was about 5 °C. Figure 14 shows the temperature variation along the marked line during the expansion stage of the thermal bubble when the applied apparent power was 6.28 VA; the temperature difference was about 20 °C.

## 6. Conclusions

In this paper, a thermal bubble-driven micropump with induction heating and heat source insulation was designed and fabricated. The experiments of the pumping flow rate and the back pressure of the heat source insulation micropump were carried out. The back pressure and the pumping flow rate were reduced compared with the micropumps of the same heating method without heat source insulation. A fluorescent temperature measurement system with microscope was built, and the fluorescent temperature calibrations of Rodamine B solutions from 21 °C to 90 °C were carried out. Then based on the temperature calibration of the Rodamine B solution, the temperature in parts of the heat insulation channel and the main pumping channel were measured. The temperature of the solution in the main pumping channel of the heat source insulation micropump was reduced compared to the temperature in the insulation channel. When the apparent power was 6.28 VA, the temperature of the pumped liquid was less than 35 °C in the main pumping channel. If the pumped liquid contained heat sensitive biological or chemical substances, the thermal bubble-driven micropump with heat source insulation can be used. For liquid without heat sensitive biological or chemical substances and high requirements for the pumping flow rate, a thermal bubble-driven micropump without heat source insulation can be adopted. In order to further reduce the heat effect of thermal bubbles on the fluid being pumped, we could increase the length of the heat insulation channel.

## Figures and Tables

**Table 1:** Performance comparison between this work and other thermal driven micropumps.

Reference	Heating Method	Dimensions of Micropump (mm^3^)	Flow Rate (µL/min)	Backpressure (Pa)	Energy Consumption (W)
Jung [17]	Resistance heating	12 × 12 × 2	8	370	3.4
Tsai [16]	Resistance heating	Not mentioned	5	377	1
Liu [18]	Induction heating	25 × 25 × 4	102	230	2.97
Yokoyama [9]	Resistance heating	50.5 × 16.5 × 1	0.9	Not mentioned	0.16
This work	Induction heating	20 × 12.5 × 3.5	28.6	118	9.04 (VA)
